# Human Fall Detection with Infrared Imaging: A Comparison of Graph Convolutional Networks and YOLO

**DOI:** 10.3390/s26092794

**Published:** 2026-04-30

**Authors:** Karol Perliński, Artur Faltyński, Aleksandra Świetlicka

**Affiliations:** Institute of Automatic Control and Robotics, Poznan University of Technology, 61-131 Poznań, Poland; karol.perlinski@student.put.poznan.pl (K.P.); falatynski.artur@gmail.com (A.F.)

**Keywords:** fall detection, infrared imaging, graph convolutional networks, YOLO, human motion analysis

## Abstract

This paper presents a comparative study of two artificial intelligence approaches—graph convolutional networks (GCNs) and the YOLO object detection algorithm—for analyzing human fall events using infrared imaging. From the AI perspective, the study introduces a GCN model that achieves over 99% classification accuracy by modeling 2D and 3D skeletal data as graph structures and evaluates the real-time detection capabilities of YOLOv8 on infrared video frames. On the engineering side, the research addresses practical challenges in elderly care and healthcare monitoring systems by demonstrating how these AI methods can accurately detect and classify fall directions under infrared conditions. The results highlight each model’s strengths and propose a hybrid framework combining YOLO’s spatial localization with GCN’s motion-pattern analysis for future real-world applications.

## 1. Introduction

Aging of society constitutes one of the key challenges for modern medicine and social care systems. As the years go by, the risk of falls increases, which can lead to serious health consequences such as fractures, head injuries, and, in extreme cases, even death. In addition to the direct physical consequences of falls, older adults often experience a persistent fear of falling, which may itself contribute to reduced mobility, activity avoidance, and further functional decline. Recent evidence in community-dwelling older adults has shown that fear of falling is significantly associated with fall history and functional balance measures, which highlights the importance of early fall-risk assessment and monitoring in this population [1]. This issue particularly affects elderly individuals, who often live alone or reside in medical care facilities. Furthermore, certain disorders, such as Parkinson’s disease, involve dopamine-based therapies that may induce adverse effects, including postural instability and dyskinesia. Several studies have reported the application of fall detection systems in such patients [2]. In such situations, a rapid response to incidents related to loss of movement stability is crucial, as it helps minimize health consequences and prevent long-term complications.

The dynamic development of technology and artificial intelligence-based systems enables effective monitoring and analysis of human movement. In particular, the use of long-wave infrared cameras in combination with artificial neural networks opens new perspectives in the field of movement stability assessment and fall detection. This approach not only enhances the precision of analysis but also allows operation in challenging lighting conditions, making it particularly useful in practice.

This study focuses on the use of graph convolutional networks (GCNs) [3] and the YOLO (You Only Look Once) algorithm [4] to analyze data recorded using long-wave infrared cameras. The goal is to develop a system capable of effectively detecting and classifying different directions of falls, which could be applied in the future in systems supporting the care of elderly individuals and patients in medical facilities.

The aim of this study is to analyze a database consisting of video recordings and corresponding data files describing human movement, particularly situations involving falls [5]. As part of the project, three main directions of falls were classified: forward, backward, and sideways. Additionally, a category for activities other than falls was included to reduce the number of false alarms in real-world system applications. The data was processed and analyzed using advanced artificial intelligence techniques, including GCN and the YOLO algorithm.

Beyond the application to healthcare monitoring, this study contributes to the broader field of pattern recognition by providing a systematic comparison between pixel-based (YOLO) and graph-based (GCN) representations for dynamic human activity analysis. Such a comparison is relevant to the community as it addresses the trade-offs between raw image-based detection and structured motion modeling.

### Motivation and Contributions

Recent fall detection studies increasingly explore privacy-friendly sensing, including thermal/infrared imagery and pose/skeleton representations.

Thermal datasets such as eHomeSeniors [6] demonstrate that infrared sensing can reduce privacy concerns while maintaining strong recognition performance. At the same time, many works focus on a single representation (either image-based detection or skeleton-based modeling), which makes it difficult to directly compare trade-offs between dense pixel-level methods and structured motion representations.

In this paper, we address this gap by benchmarking two complementary paradigms on the same long-wave infrared dataset with synchronized skeletal annotations: (i) a pixel-based object detection approach (YOLOv8) operating directly on infrared frames, and (ii) a graph-based classifier (GCN) operating on 2D/3D joint graphs.

Our main contributions are:

- Unified comparison on infrared data: We provide a systematic, apples-to-apples evaluation of YOLO and GCN for fall-direction recognition under infrared conditions, enabling a direct comparison between pixel-based detection and graph-based skeletal modeling.
- Deployment-oriented analysis: We discuss practical requirements and limitations of both paradigms (e.g., dependence on pose availability for GCN vs. viewpoint/occlusion sensitivity for YOLO), including implications for real-time monitoring.
- Hybrid design rationale: Based on the observed strengths, we outline a hybrid framework that combines YOLO’s fast spatial localization with GCN’s motion-pattern reasoning to improve robustness in future real-world deployments.

The article is organized as follows:

- Section 2: Related Work—We describe existing approaches to fall detection, categorizing them into vision-based, sensor-based, and hybrid systems, and highlighting their respective advantages and limitations.
- Section 3: Methodology—We present the dataset used in the study, including details on infrared recordings and skeletal data. We also explain the implementation of two fall detection methods: graph convolutional networks (GCNs) and the YOLO detection algorithm.
- Section 4: Results and Discussion—We report experimental results obtained from both methods, comparing their performance based on accuracy metrics and discussing their strengths and limitations in the context of real-world applications.
- Section 5: Conclusion—We summarize the key findings of the study and propose a potential hybrid approach combining GCN and YOLO. Future research directions are also discussed.

## 2. Related Work

There are three main types of human fall detection systems:

- Vision-based systems utilize cameras and image processing techniques to detect falls. These systems are non-invasive and can be highly accurate. For instance, the use of convolutional neural networks (CNNs) and optical flow methods in multi-camera setups has shown high accuracy in detecting falls by analyzing the relative motion between consecutive images [7,8]. Another approach involves using human skeleton estimation from video feeds to detect falls, which allows for monitoring multiple individuals simultaneously in real environments [9].
  Early vision-based methods also modeled silhouette dynamics and event causality; for example, Liao et al. employed a Bayesian belief network to distinguish slip-only events from actual falls in monocular video [10].
- Wearable systems remain one of the most widely adopted approaches for fall detection. Islam et al. [11] provided a comprehensive review of sensor-based methods, emphasizing their portability and ability to capture real-time motion data. Nooruddin et al. [12] introduced an IoT-based device-independent framework that demonstrates how such systems can adapt to different hardware platforms while maintaining detection accuracy. Mao et al. [13] later proposed a highly portable platform integrating accelerometers and gyroscopes, improving usability in everyday life.
  Although effective, these wearable methods require users to continuously carry or attach the device, which can reduce long-term compliance and limit their applicability in elderly care scenarios.
  Non-wearable sensors also include ambient sensors (e.g., WiFi and cameras) installed in the environment. WiFi-based systems use channel state information (CSI) to detect falls without requiring the user to wear a device, offering high accuracy and allowing natural daily activities [14,15]. Camera-based systems use vision and machine learning to recognize fall patterns, providing wide-area coverage but raising privacy concerns [16,17,18].
- Hybrid systems combine multiple methodologies, such as integrating vision-based and sensor-based approaches, to enhance detection accuracy and reliability [11,19]. For example, using floor vibrations in conjunction with machine learning algorithms has been proposed as a novel method to detect falls and distinguish between different fall postures [20]. Additionally, the use of re-parameterization and feature enhancement techniques in neural networks has been shown to improve detection accuracy and speed [21].

In recent years, multimodal approaches to fall detection have gained attention, as they aim to combine complementary data sources and thus increase system robustness. For example, Jiao et al. [22] introduced a multimodal fall detection framework for solitary individuals, published in Pattern Recognition (2024), which leverages multiple sensing modalities to achieve high detection accuracy in complex environments. Their results highlight the potential of combining different input representations to mitigate the limitations of single-sensor systems. In contrast, our study focuses on infrared imaging and skeletal modeling, which, while unimodal, offer significant advantages in terms of privacy preservation and robustness to lighting conditions. This distinction underlines the novelty of our approach, as it emphasizes contactless monitoring with a reduced computational footprint, while still showing strong performance in the evaluated infrared fall-detection setting.

A great overview on human fall detection using pose estimation can be found in [23]. The article presents a vision-based method for human fall detection using video frames and advanced pose estimation techniques, including transformer-based models. It emphasizes the advantages of this approach over wearable sensors and demonstrates high accuracy on several benchmark datasets such as UR-Fall, UP-Fall, and Le2i.

In recent years, the integration of fall detection with human–robot interaction (HRI) has gained significant attention, especially in the development of intelligent assistive robots for elderly care. These systems not only aim to detect falls but also interact with users in a supportive and adaptive manner.

One line of research focuses on using mobile robots equipped with visual and sensor-based systems capable of human following and fall detection. For example, Seneviratne et al. [24] developed a healthcare companion robot that combines indoor tracking, fall detection, and real-time assistance for elderly and differently abled individuals. Similarly, Zhao et al. [25] proposed a safe and compliant non-contact interaction model for walking aid robots, enabling them to detect unstable gaits and potential falls without constraining the user’s movement.

The article [26] introduces a novel fall detection system that uses videos from two camera angles and a human-like doll to simulate falls with and without head injuries, addressing the lack of datasets with occlusions and head trauma. It employs a multi-input neural network with ConvLSTM modules and head trajectory analysis to accurately detect falls and head injuries, achieving high F1 scores across multiple datasets.

In the paper [27], the author emphasizes that HRI aims not only to improve robot–human cooperation but also to train robots to pay more and more attention to humans, their emotions, their movements, and safety.

In fall detection systems, deep learning approaches have become increasingly prominent due to their ability to process complex and high-dimensional data. Islam et al. [28] provided a comprehensive review of deep learning-based systems, showing how CNN, LSTM, and autoencoder architectures have been applied to fall detection with strong accuracy across benchmark datasets. Villa and Casilari [29] proposed an energy-efficient system that integrates LoRa communication with hybrid algorithms, demonstrating how deep models can be optimized for low-power deployment in wearable and IoT contexts. Toosizadeh et al. [30] presented a radar-based survey of fall detection techniques, highlighting the potential of non-vision modalities combined with deep learning to achieve robust, contactless monitoring. These works illustrate how deep architectures and hybrid strategies can enhance recognition performance while addressing different practical constraints, from computation efficiency to sensing modality.

Traditional machine learning methods remain widely used, especially in systems relying on wearable sensors like accelerometers and gyroscopes. Algorithms such as support vector machines (SVMs), k-nearest neighbors (kNN), decision trees, and logistic regression are employed to classify fall events based on extracted features. Ensemble methods, which combine multiple classifiers, have shown superior performance in terms of accuracy and robustness compared to individual models. These techniques are often chosen for their efficiency, interpretability, and effectiveness in real-time applications with moderate computational constraints [30,31].

In contrast, heuristic and threshold-based methods provide a lightweight solution for fall detection [32,33]. These approaches involve simple rule-based logic, such as double thresholding or fuzzy logic, applied to basic features derived from sensor data. By setting specific thresholds for parameters like acceleration or body orientation, these methods can reliably identify falls with low computational overhead. Although less adaptive than machine learning models, they offer fast response times and are well-suited for real-time deployment in resource-constrained environments such as wearable or mobile devices.

Graph neural networks (GNNs) have also shown promise in enhancing human behavior recognition in HRI settings. In [34], the authors explored the use of GNNs to recognize non-verbal social behaviors, which can complement fall detection by improving contextual understanding of human motion. In related work, ref. [35] demonstrated the application of GNNs in natural language processing for HRI, showing the versatility of graph-based models in multimodal human–robot communication.

The proposed solution in this study introduces a novel combination of infrared imaging and artificial intelligence techniques for fall detection, setting it apart from previous approaches found in the literature. In contrast to the method described in e.g., [36], which relies on inertial measurement unit (IMU) sensors and a graph convolutional network (GCN) with variable time windows (T-GCN), our approach utilizes infrared video data, enabling fully contactless monitoring. This is particularly important in healthcare settings where comfort and hygiene are critical. While ref. [36] enhances temporal modeling through adaptive time windows, it operates solely on IMU data and evaluates its performance on a small, manually constructed dataset. Our method, on the other hand, integrates both skeletal modeling and visual data analysis, allowing for more comprehensive and scalable monitoring. Recent work by A. Flaborea et al. has shown that contracting skeletal kinematics is effective for human-related video anomaly detection [37], reinforcing the value of skeleton-centered representations that our GCN-based pipeline also exploits.

Moreover, the system presented in this paper differs from the advanced graph-based spatial-temporal convolutional and attention network (GSTCAN) described in [38], which focuses on high-accuracy classification using attention-enhanced GCNs. Although the authors in [38] demonstrate excellent performance of their solution across multiple datasets, it is designed primarily for controlled experimental conditions and does not consider real-time deployment using infrared sensors. Our study addresses this gap by incorporating the YOLOv8 model for efficient spatial localization of the human figure and potential fall events, which is then complemented by GCN-based motion analysis for fall classification. This dual-model framework enhances both the responsiveness and interpretability of fall detection.

A directly relevant reference for the present study is the recent work of Gutiérrez et al. on fall detection in low-illumination environments from far-infrared images using pose detection and dynamic descriptors. That study introduced FIR-Human as the first large public FIR dataset of its kind and evaluated an FIR-based fall detection pipeline built on pose estimation and dynamic descriptors. In their reported FIR-Human evaluation, the best-performing configuration (ViTPose) achieved 95.83% sensitivity, 96.92% specificity, and 96.63% accuracy, providing an important published reference point for studies using this dataset. Unlike that pipeline, our work focuses on a comparative analysis between graph-based skeleton modeling (GCN) and image-based detection (YOLOv8) under a unified infrared benchmark [39].

A broader perspective is provided by recent literature reviews and movement-analysis studies. The recent survey by Wastupranata et al. highlights that deep-learning-based abnormal human behavior detection, including fall detection, remains sensitive to factors such as image quality, camera viewpoint, and confusion with visually similar actions [40]. In addition, a recent 2026 study on postural stability dynamics demonstrated that quantitative nonlinear movement analysis can distinguish stable and unstable gait conditions with classification accuracy above 90%, reinforcing the relevance of motion-centered descriptors for safety-related human movement assessment [41]. Although these works are not directly comparable to our FIR-Human fall-direction benchmark, they help position our study within the broader literature on abnormal behavior analysis and human movement assessment.

Additionally, by leveraging thermal imaging, we mitigate issues related to privacy, lighting variability, and occlusion. This makes our system not only technically innovative but also more suitable for deployment in real-life healthcare and elderly care facilities, where continuous, non-invasive observation is essential.

These studies highlight the growing role of HRI in fall detection, where robotic systems are no longer passive observers but active participants in ensuring user safety, autonomy, and well-being.

## 3. Methodology

### 3.1. Data Acquisition and Preprocessing

#### 3.1.1. Dataset Availability and Recording Setup

We use the FIR-Human dataset [5], which is publicly available for academic and research use under the IEEE DataPort license. FIR-Human contains over 250,000 far-infrared (FIR) images with synchronized 2D and 3D annotations of 19 main body joints for each frame. The dataset was recorded in a laboratory environment using a FIR camera synchronized with a high-speed motion-capture system, which provides accurate 3D joint coordinates and their 2D projections onto the image plane. The original dataset publication does not provide detailed information about the manufacturer, model, or origin of the recording equipment. The FIR recordings were captured at 23.98 FPS with a spatial resolution of 480×640 pixels. To increase diversity, subjects wore different clothing, and the laboratory thermal conditions varied between recordings (reported temperature range: 16–31 °C).

#### 3.1.2. Subjects and Demographics

The dataset includes five volunteers (four males and one female) with BMI ranging from 16 to 24. The reported age range spans from 25 to 56 years, and heights range from 161 to 180 cm. All participants provided informed consent as reported by the dataset authors.

#### 3.1.3. Actions, Views, and Dataset Structure

FIR-Human contains 27 action classes: 26 daily-life activities and one fall class. Actions are recorded from multiple viewpoints (four subject/camera positions enabling frontal, rear, and side perspectives). The dataset is organized into three blocks: (i) a training block containing daily-life activities performed by four volunteers; (ii) a validation block containing a different set of activities performed by one volunteer; and (iii) a fall block containing forward, backward, and side falls performed by four volunteers from different perspectives.

#### 3.1.4. Fall Taxonomy (Static/Dynamic/Slow)

According to the dataset description, falls can start from static or dynamic situations, and a subset corresponds to low-energy slow falls. In our work, we follow the original dataset labeling and interpret these categories as: static (fall initiated from a stationary posture), dynamic (fall initiated during motion, e.g., gait/transition), and slow (controlled low-energy descent with longer fall duration).

#### 3.1.5. Skeletal Annotation Format (2D/3D Matrices)

The three-dimensional matrix refers to a coordinate system with its origin at ground level, while the two-dimensional matrix corresponds to a coordinate system with its origin positioned in one corner of the image. The three-dimensional matrix has dimensions x×19×3, where

- *x*—the number of frames (variable depending on the duration of each video sequence);
- 19—the number of key body points (joints);
- 3—the number of spatial dimensions in the given data (*x*, *y* and *z*).

**Figure 1:**
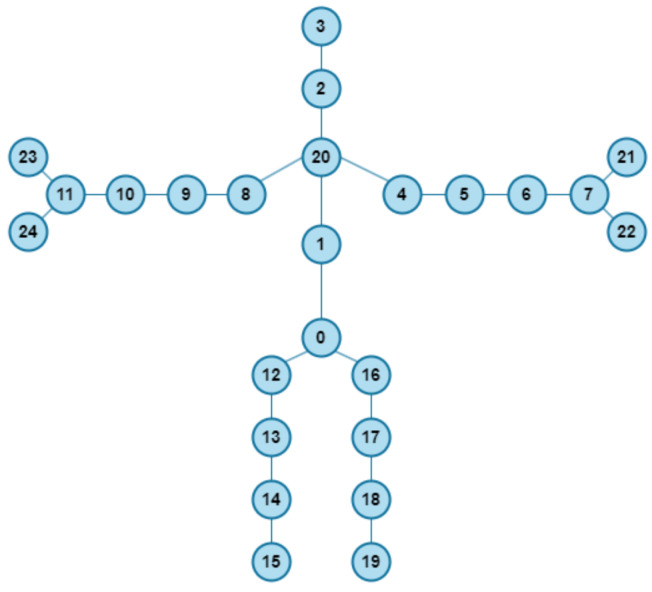
Sample skeletal data representation with annotated body joints. The numbering follows the original convention used in [34].

For example, the value at position (320, 17, 2) of such a matrix represents the *y*-coordinate of the right ankle in the 320th frame. The same system applies to the two-dimensional matrix, with only the difference being the dimensions of said matrix, which are x×19×2, because we operate only on two dimensions (*x* and *y*). The labeling of joints used in this study is presented in Table 1, while an example of skeletal data visualization is shown in Figure 1.

#### 3.1.6. Sequence Characteristics and Fall Recording Protocol

In the analyzed dataset, the number of frames per fall recording ranged from 140 to 350. The recorded falls included three types: forward, backward, and sideways. Each type of fall was further classified into three variants: static, dynamic, and slow. For each type and variant of fall, two recordings were made, resulting in a diverse and comprehensive dataset for analysis.

**Figure 2:**
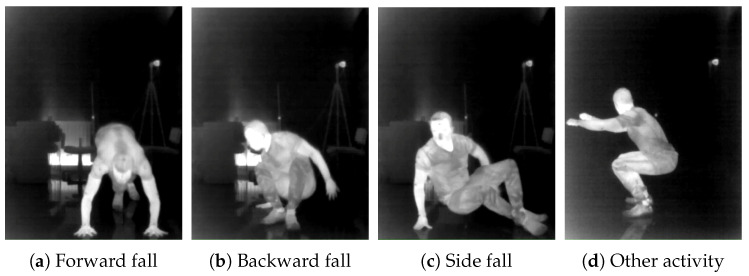
Example FIR frames from the FIR-Human dataset illustrating fall directions and a non-fall activity.

Figure 2 shows representative FIR frames illustrating the three fall directions and an example non-fall activity.

Table 2 summarizes the key properties of the FIR-Human dataset used in this study.

**Figure 3:**
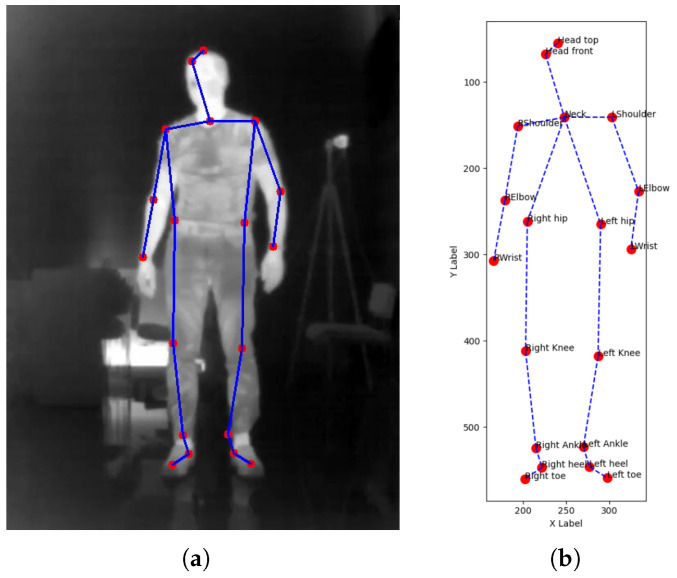
(**a**) A sample frame with annotated body joints and their connections. Red dots indicate joint positions, while blue solid and dashed lines represent connections between joints. (**b**) Skeletal data represented as a 2D graph.

Based on the collected data, a graph database was created for training the graph convolutional network (GCN), where each frame of joint positions was converted into a graph structure. In total, 189,663 graphs were constructed, representing various fall directions and movement patterns. Of them, 132,764 were training graphs, 28,449 validation graphs, and 28,450 test graphs. Simultaneously, video recordings were used to train the YOLO model for object detection directly from infrared image frames. This dual preprocessing approach allowed the development of two independent fall detection systems, each leveraging different aspects of the dataset. Figure 3 illustrates the joint annotations and their graph-based representation used for GCN input.

### 3.2. Graph Convolutional Networks (GCNs)

Graph convolutional networks (GCNs) [3] are a specialized type of graph neural networks (GNNs) that extend the concept of classical convolutions from regular data, such as images, to data represented in the form of graphs. Unlike convolutional neural networks (CNNs) for images, which operate on pixels arranged in a fixed grid, GCNs enable convolutional operations on graphs with arbitrary structures. In GCNs, the convolution operation on a graph involves aggregating information from a node’s neighbors.

The following equation describes the output of a single graph convolutional layer in the GCN architecture:

(1) $$h_{v}^{\left(k+1\right)}=\sigma \left(\sum_{u\in N(v)}\frac{1}{c_{vu}}W^{\left(k\right)}h_{u}^{\left(k\right)}\right),$$

where $h_{v}^{\left(k\right)}$ denotes the feature vector of node *v* at layer *k*, $h_{u}^{\left(k\right)}$ denotes the feature vector of a neighboring node *u* at layer *k*, N(v) represents the set of neighbors of node *v*, $c_{vu}$ is a normalization constant, $W^{\left(k\right)}$ is the trainable weight matrix at layer *k*, and σ(·) denotes the activation function (e.g., ReLU).

#### 3.2.1. GCN Model Architecture and Training Protocol

##### Graph Construction and Node Features

Each video frame is converted into a single graph with N=19 nodes corresponding to body joints. We use the 3D joint coordinates from *data_3D* as node features, i.e., each node is represented by a 3D vector (x,y,z). Graphs are generated frame-by-frame; therefore, the total number of graphs equals the total number of frames in the selected recordings. In our implementation, the graph connectivity is fully connected, i.e., edges are defined between all pairs of distinct joints. During training, we apply a simple edge-perturbation augmentation by randomly removing a fraction of edges. During inference, graphs can be created without ground-truth labels (i.e., label = None).

The fully connected graph topology was deliberately chosen as a simple relational baseline for the comparative study. In this representation, each joint can exchange information directly with every other joint, which facilitates modeling of long-range inter-joint dependencies within a frame-level GCN. This can be useful for fall analysis, where the relevant motion pattern may involve global body configuration changes rather than only local anatomical interactions.

At the same time, we acknowledge that this graph definition is not explicitly biomechanical. An anatomical graph based on natural skeletal connections (e.g., shoulder–elbow–wrist or hip–knee–ankle) would provide a stronger physical prior and could improve interpretability by constraining message passing to physically meaningful body links. In contrast, the fully connected design is more flexible but less interpretable, because it includes both anatomical and non-anatomical relations. Since no dedicated ablation between fully connected and anatomical graph topologies was performed in the present study, we treat the current choice as a design decision of the baseline model rather than as evidence of superiority over biomechanically grounded alternatives.

##### Partitioning Protocol and Subject Overlap

For the GCN experiments, recordings were first converted into frame-level graph samples, and the resulting graphs were then partitioned into training, validation, and test subsets. This protocol did not enforce subject-wise separation; therefore, samples originating from the same volunteers could appear in more than one subset. Consequently, the reported results should not be interpreted as a subject-independent evaluation. We note that FIR-Human contains only a limited number of subjects for the fall-direction task, which constrains the feasibility of a statistically stable subject-wise benchmark within the current dataset.

##### Network Architecture

The proposed GCN classifier consists of four graph convolution layers implemented with GCNConv and residual (skip) connections. The layer widths are 3→32→64→128→256. After each graph convolution, we apply batch normalization and ReLU activation. A global mean pooling operation aggregates node embeddings into a graph-level descriptor, followed by a three-layer MLP head: 256→128→64→4. Dropout with p=0.5 is applied before and within the MLP head. The network outputs class scores for four classes (*Forwards*, *Backwards*, *Side*, and *Other*).

##### Training Setup and Hyperparameters

We train the model using the Adam optimizer with learning rate $1\cdot 10^{-3}$ and weight decay $1\cdot 10^{-4}$. The batch size is 32 graphs. To improve convergence, we use a StepLR scheduler that reduces the learning rate every 20 epochs by a factor of 0.5. Gradient norm clipping is applied with a maximum norm of 2.0. We use a weighted cross-entropy loss to account for class imbalance, with class weights [1.0,2.0,3.0,1.0] for (*Forwards*, *Backwards*, *Side*, and *Other*). We use training runs for up to 200 epochs with early stopping (patience = 10) based on validation accuracy.

##### Temporal Information Handling

The GCN backbone performs frame-level graph classification, i.e., each frame is treated as an independent graph that inherits the action label of the corresponding sequence during training. We do not use temporal edges, sequence windows, or spatio-temporal GCN variants. To obtain a single decision for a complete recording at inference time, we aggregate frame predictions across the sequence. We use two aggregation strategies: (i) *mean_softmax* (default), i.e., averaging the softmax probabilities over frames, and (ii) *majority_vote*, i.e., voting over per-frame predicted classes. The same aggregation can be applied during evaluation to report sequence-level metrics in addition to frame-level metrics. In future work, we plan to extend the approach toward explicit temporal modeling (e.g., spatio-temporal GCNs or learned sequence-level aggregation).

##### Partitioning Protocol

For the GCN experiments, each recording was first converted into frame-level graph samples, and the resulting graphs were then partitioned into training, validation, and test subsets. Thus, the adopted protocol is frame-based rather than sequence-based, and temporally related frames from the same original recording may appear in different subsets. As a consequence, the reported GCN results should be interpreted as performance under the adopted frame-level evaluation protocol rather than as a strictly sequence-independent estimate of generalization.

All GCN training and inference settings are summarized in Table 3.

#### 3.2.2. GCN in Analyzing Human Falls

In the performed research, GCNs are used to process skeletal data, which is represented as a graph:

- Nodes: Human body joints (e.g., elbow, wrist).
- Edges: Defined using a fully connected topology rather than only anatomical body links. Thus, each joint is connected to all remaining joints in the graph.

This representation enables the model to capture both local and global relational patterns, although it is less directly interpretable in biomechanical terms than an anatomical skeleton graph.

Each node is described by a feature vector, which may include:

- Spatial coordinates of the joint (e.g., x,y,z).
- Other dynamic features, such as joint velocity or acceleration.

Application of GCN in the project:

- GCNs enable the propagation of information between body joints, allowing the model to consider both local and global dependencies in movement patterns.
- Graph convolutional layers allow the model to “learn” joint representations in the context of their surroundings, which is crucial for motion analysis and recognizing movement patterns.
- The introduction of graph convolution enables the modeling of interactions between different body parts (e.g., dependencies between hand movement and torso motion).

**Figure 4:**
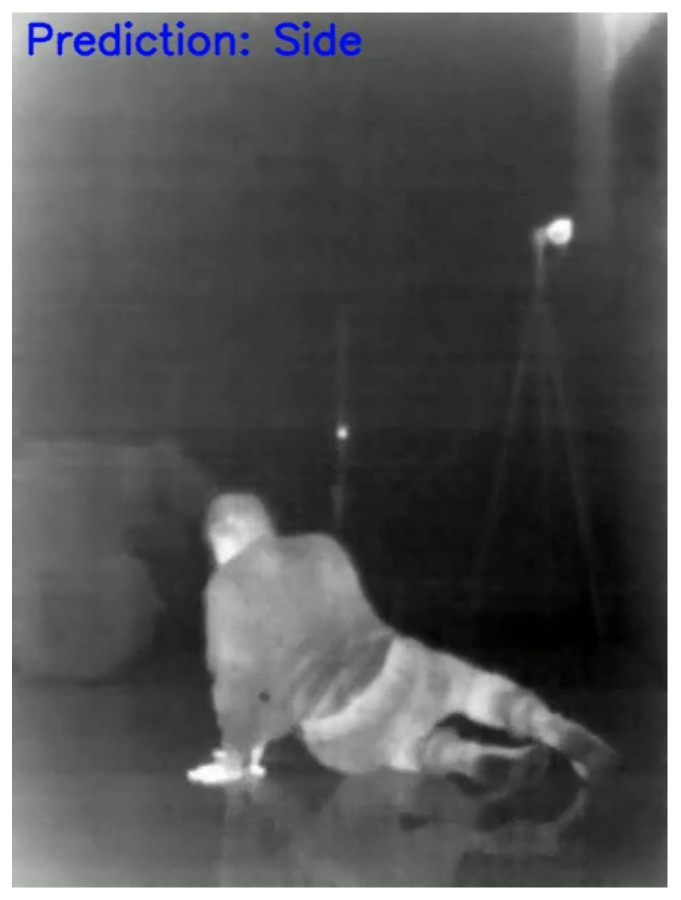
Visualization of side fall prediction using graph convolutional networks.

The application of GCN for side fall detection is visualized in Figure 4, shown as the blue text in the top left corner of the image.

### 3.3. Problem Description: Fall Detection Using Graphs

Fall detection using graphs is an advanced task in human motion analysis that leverages graph structures to represent skeletal data and classify physical activities. This problem involves not only the identification of fall events but also their effective differentiation from other activities that may look similar to the algorithm, which minimizes the risk of generating false alarms.

The primary goal of fall detection is to accurately distinguish between fall events and other movements. A fall is characterized by a specific motion pattern, often involving:

- Sudden changes in body position in space;
- Dynamic movements, such as a rapid lowering of the body’s center of mass;
- Unusual movement trajectories compared to typical daily activities.

However, in practical scenarios, many activities (e.g., squatting, bending over, or playing dynamic sports) may resemble falls, thereby increasing the likelihood of false positives. Therefore, a critical component of the model is its ability to correctly classify both falls and non-fall activities.

To reduce the number of misclassifications, the model includes an additional class labeled *Other*, which is designed to capture all activities unrelated to falls. The inclusion of this class provides several benefits:

- The neural network more effectively distinguishes falls from other types of movement.
- False alarms triggered by fall-like movements (e.g., abrupt or intense motion) are significantly reduced.

Adding this class required extending the dataset with additional video sequences depicting a variety of human actions, such as walking, running, squatting, or lifting objects. This enabled the model to learn a broader and more diverse range of motion patterns, improving its robustness in real-world applications.

### 3.4. YOLO for Object Detection

YOLO [4] is an image detection model that processes the entire image in a single pass, allowing it to simultaneously detect and localize objects with exceptional speed. We used the v8 version of YOLO, as well as the CUDA platform.

In this project, YOLO was used as a key component of the object detection system for video data. Its primary role is to precisely detect human movement in real time. The algorithm was applied to the final few frames of each video sequence to detect the human subject and extract key bounding box information. The method divides the image into a grid, where each cell predicts bounding boxes and class probabilities. This simultaneous detection and localization approach ensures real-time performance, which is crucial for timely fall detection. By accurately identifying the region containing the human figure in video frames, YOLO enhances the efficiency and reliability of the system.

**Figure 5:**
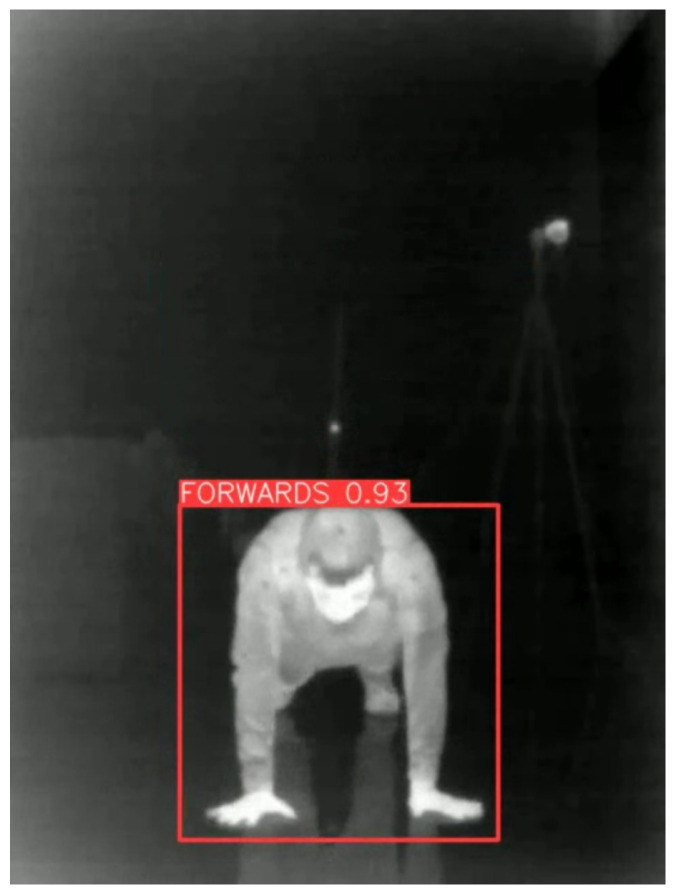
Schematic of the YOLO detection mechanism showing bounding-box prediction.

The YOLO detection mechanism and bounding-box prediction are shown schematically in Figure 5. The red bounding box indicates the region detected by the algorithm, with the corresponding class prediction displayed above the box.

#### YOLOv8 Training Protocol and Implementation Details

We trained a YOLOv8 detector using the Ultralytics implementation with the YOLOv8m variant (model = yolov8m.pt). The model was initialized from pretrained weights (pretrained = true) and fine-tuned on infrared frames. Training was performed for 150 epochs with batch size 16 and input image size imgsz = 640. For reproducibility, we used a fixed seed (seed = 0) and enabled deterministic execution (deterministic = true). Mixed precision was enabled (amp = true).

The optimizer was set to auto, i.e., the Ultralytics pipeline selected the optimizer configuration automatically; the base hyperparameters used in our run were lr0 = 0.01, lrf = 0.01, momentum = 0.937, weight_decay = 0.0005, and a warmup of 3 epochs (warmup_epochs = 3.0). Data augmentation followed the Ultralytics training recipe with HSV jitter (hsv_h = 0.015, hsv_s = 0.7, hsv_v = 0.4), geometric transforms (translate = 0.1, scale = 0.5), horizontal flipping (fliplr = 0.5), and mosaic augmentation (mosaic = 1.0) disabled for the last 10 epochs (close_mosaic = 10). MixUp and copy–paste augmentations were disabled (mixup = 0.0, copy_paste = 0.0).

During evaluation and confusion-matrix generation, non-maximum suppression used an IoU threshold of iou = 0.7; the confidence threshold was left at the Ultralytics default (conf = null). All YOLOv8 training and inference settings are summarized in Table 4.

To ensure a fair comparison, we report YOLOv8 results obtained from a fixed configuration (Table 4) and GCN results obtained from the fixed architecture and training recipe (Table 3), both evaluated on the same dataset split.

## 4. Results and Discussion

Experimental evaluations were conducted on a dataset comprising infrared recordings with annotated fall events. The GCN-based classifier achieved an accuracy above 95% across all fall directions. Meanwhile, the YOLO approach showed competitive performance in detecting the human subject and localizing relevant image regions. Key performance metrics, such as the training loss and accuracy progression, are summarized in the graphs below.

### 4.1. Unified Evaluation Metrics

To enable a direct comparison between the graph-based classifier (GCN) and the image-based detector (YOLOv8), we report a unified set of classification metrics for fall-direction recognition, including per-class precision, recall, and F1-score. For the GCN, the model operates on frame-wise graphs; therefore, each frame is first classified independently, and frame-level results are reported. All frame-level metrics reported in this study, including confusion-matrix-based analyses, were computed on a held-out frame-level test split. However, since the partitioning was performed at the frame level, frames originating from the same recording could appear in different subsets. Therefore, the reported test results should be interpreted as independent at the frame level, but not as a fully independent sequence-level evaluation. In addition, we provide a complementary sequence-level analysis, in which frame-level outputs belonging to the same recording are aggregated into a single predicted label using either *mean_softmax* or *majority_vote*. Since the underlying split was performed at the frame level, these aggregated sequence-level results are reported as supplementary evidence rather than as a strictly leakage-free sequence-wise benchmark. To further illustrate this aggregation step, we additionally report sequence-level confusion matrices for both aggregation strategies. For YOLOv8, we additionally report standard detection metrics, namely mAP@0.5 and mAP@0.5:0.95, using the default Ultralytics validation procedure.

### 4.2. GCN Results

**Figure 6:**
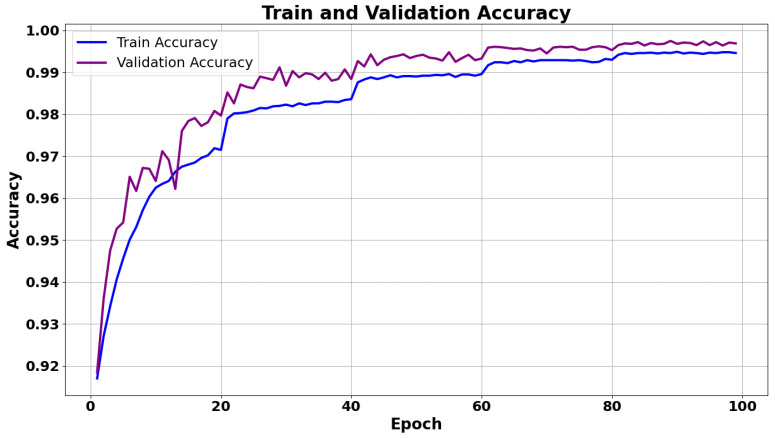
Training and validation accuracy curves (GCN) over 100 epochs.

Figure 6 presents the training and validation accuracy of the GCN model. Noticeable drops in the training accuracy curve at every 20 epochs correspond to the StepLR scheduler reducing the learning rate by a factor of 0.5 at each 20-epoch step, which momentarily limits weight updates and induces temporary fluctuations. These fluctuations are a natural consequence of using a learning rate schedule. The purpose of reducing the learning rate is to allow the model to fine-tune the weights more carefully in later stages of training. The validation accuracy quickly rises in the early epochs and then stabilizes around 99.5%, indicating strong generalization to unseen data.

**Figure 7:**
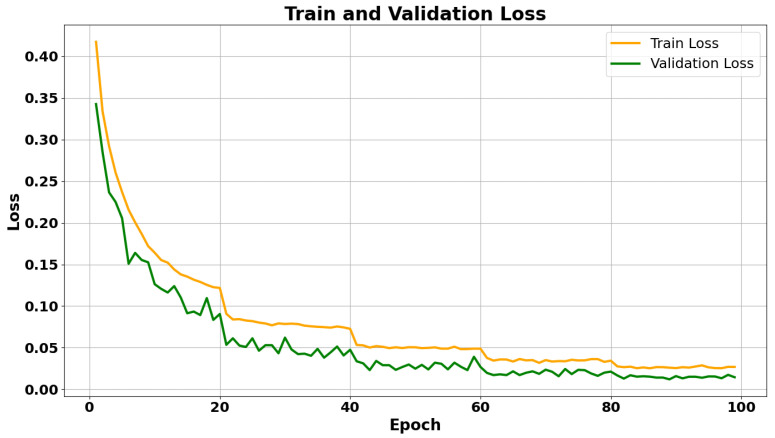
Training and validation loss curves (GCN) over 100 epochs.

Figure 7 shows the training and validation loss curves. Similar periodic drops appear in the training loss at the same 20-epoch intervals due to the learning rate schedule. Overall, the validation loss decreases steadily and converges below 0.02, demonstrating effective error minimization without signs of overfitting throughout training.

**Figure 8:**
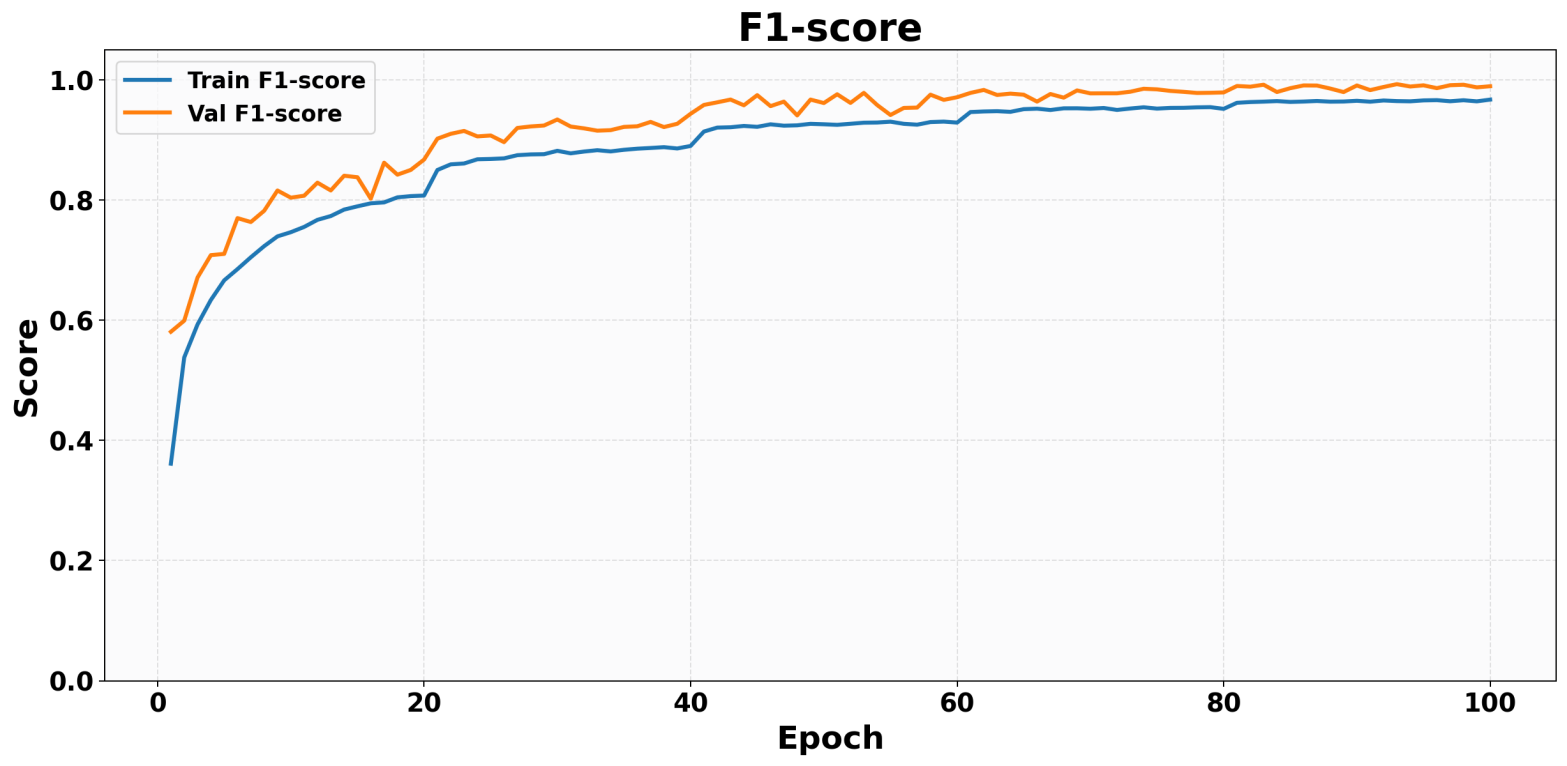
Training and validation F1-score curves (GCN) over 100 epochs.

Figure 8 presents the evolution of the training and validation F1-score during GCN training. Both curves increase rapidly in the early epochs, indicating that the model quickly improves the balance between precision and recall. The training F1-score rises from approximately 0.36 at the beginning of training to nearly 0.97 in the final stage, while the validation F1-score increases from about 0.58 to almost 1.00. The validation curve remains slightly above the training curve for most of the training process, which is consistent with the strong generalization performance observed in the other evaluation plots. After the initial growth phase, both curves gradually stabilize, suggesting convergence of the classifier and robust recognition of fall-direction classes.

**Figure 9:**
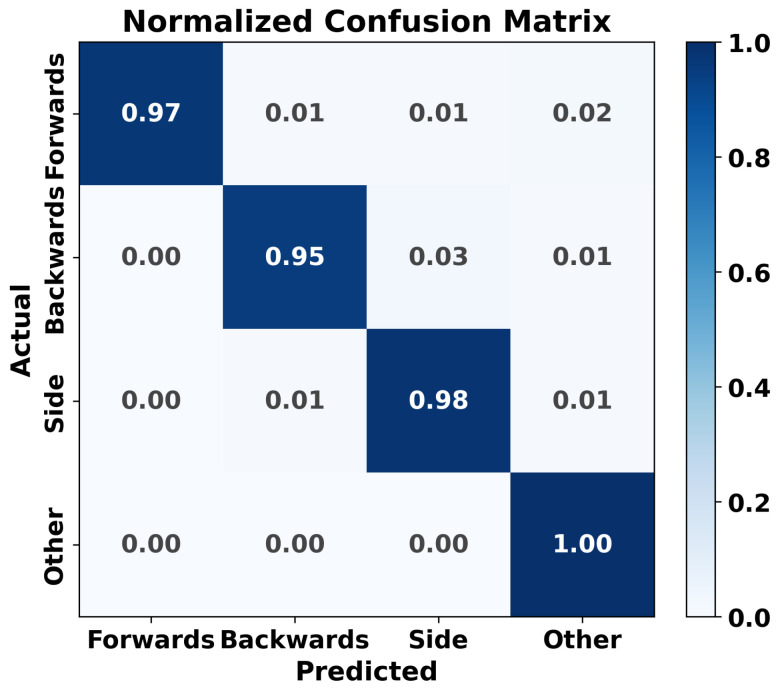
Normalized confusion matrix for the GCN classifier.

In Figure 9 the normalized confusion matrix for the GCN classifier is presented. The matrix shows the performance of the model in predicting four classes: “Forwards,” “Backwards,” “Side,” and “Other.” The diagonal elements indicate the proportion of correct predictions for each class, with high values, such as 0.97 for “Forwards” and 0.98 for “Side,” demonstrating strong classification accuracy. Off-diagonal elements are minimal, suggesting that misclassifications are rare. Overall, the classifier exhibits high performance, particularly in distinguishing between the “Forwards” and “Backwards” categories, with the “Other” class being perfectly identified (1.00).

### 4.3. YOLO Results

**Figure 10:**
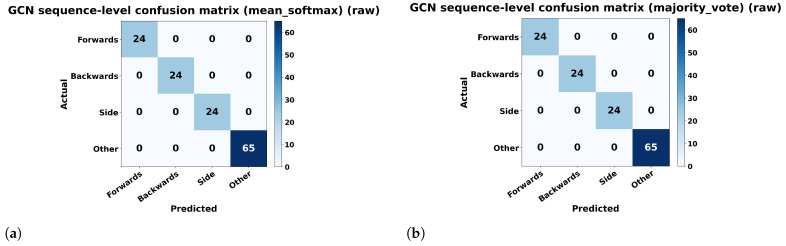
Sequence-level confusion matrices for the GCN model obtained by aggregating frame-level predictions over entire recordings using two strategies: (**a**) *mean_softmax* and (**b**) *majority_vote*.

To complement the frame-level evaluation, we also analyzed sequence-level predictions obtained by aggregating frame-wise GCN outputs within each recording. Two aggregation strategies were considered: *mean_softmax*, where the softmax probabilities are averaged across all frames of a sequence, and *majority_vote*, where the final label is determined by voting over frame-level predictions. The resulting sequence-level confusion matrices are shown in Figure 10. In both cases, the matrix is perfectly diagonal, indicating that all evaluated sequences were correctly classified into the four classes (*Forwards*, *Backwards*, *Side*, and *Other*). These results suggest that aggregating frame-level predictions can further stabilize the final decision at the recording level. At the same time, because the split was performed at the frame level, these sequence-level results should be interpreted as supplementary evidence rather than as a fully independent sequence-level benchmark.

Precision (*P*) and recall (*R*) are standard metrics for evaluating detection models. Precision is defined as the ratio of true positive detections to all positive predictions:

(2) $$P=\frac{\mathrm{TP}}{\mathrm{TP}+\mathrm{FP}},$$

where TP denotes true positives and FP false positives. Recall measures the ratio of true positives to all actual positives:

(3) $$R=\frac{\mathrm{TP}}{\mathrm{TP}+\mathrm{FN}},$$

with FN denoting false negatives.

**Figure 11:**
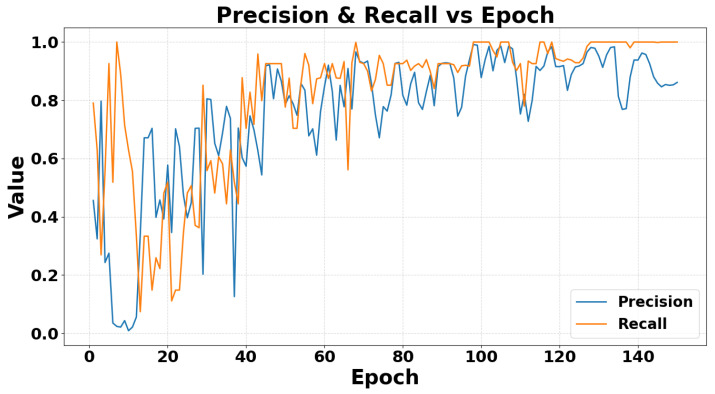
A chart showing the change in the precision and recall metric during the model validation process.

Figure 11 shows how *P* and *R* evolve over 150 training epochs. After an initially unstable warmup phase, both metrics steadily improve: recall quickly rises into the high-0.8 to 1.0 range, while precision converges above 0.85, indicating that the model becomes both sensitive to actual events and accurate in its positive predictions.

The classification loss (cls loss) is the cross-entropy error between the predicted class probabilities and the true labels, measuring how well the network distinguishes fall from non-fall events. The bounding-box regression loss (box loss) is an IoU-based error that quantifies the discrepancy between predicted and ground-truth bounding boxes, reflecting the model’s localization precision. The distribution focal loss (DFL loss) is an auxiliary term that reshapes the box regression target distribution to emphasize harder-to-predict offsets, smoothing the optimization landscape.

**Figure 12:**
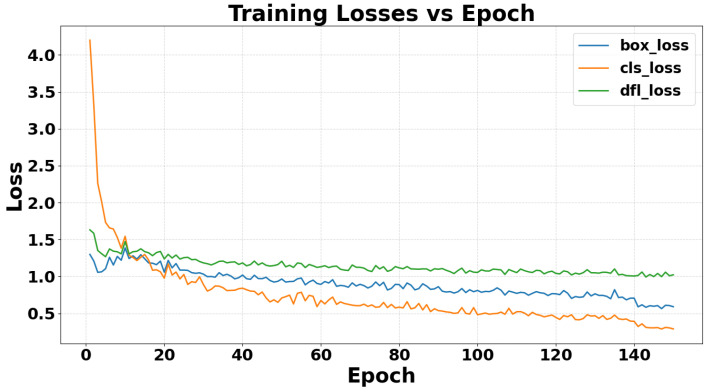
A chart showing the change in the train classification loss function during model training.

Figure 12 shows the evolution of these three losses over 150 epochs. The cls loss (orange curve) exhibits the steepest initial decline, dropping most sharply in the first 20–30 epochs before tapering off. The box loss (blue curve) decreases more gradually, indicating steady improvement in bounding-box accuracy. The DFL loss (green curve) closely follows the box loss trend but with reduced fluctuations, promoting stable convergence in later epochs. All three curves display early fall followed by a plateau, with cls loss showing the most pronounced early drop among the three.

**Figure 13:**
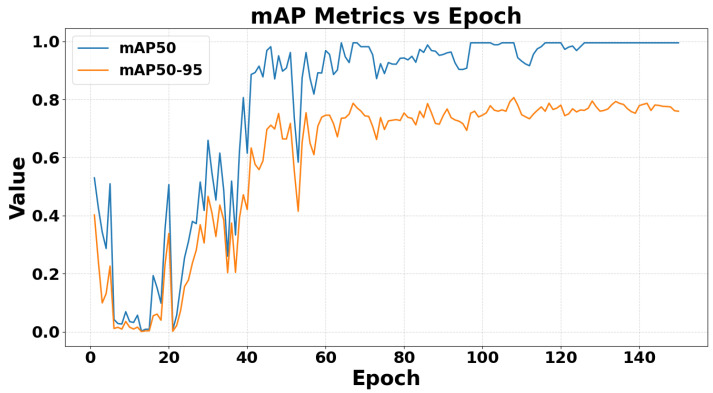
A chart showing the evolution of mAP@0.5 and mAP@0.5:0.95 during YOLO model training and validation.

The mean average precision at IoU 0.5 (mAP@0.5) and the stricter mAP@0.5:0.95 are shown in Figure 13. In the early training stage, both metrics exhibit noticeable fluctuations, which is typical of the initial optimization phase. Starting from approximately epoch 25, both curves show a clear upward trend, indicating rapid improvement in detection quality. The mAP@0.5 metric increases sharply and exceeds 0.9 after roughly 40–45 epochs, then remains close to 1.0 for most of the later training stage. In contrast, mAP@0.5:0.95 improves more gradually and stabilizes at approximately 0.75–0.79, reflecting the more demanding evaluation across multiple IoU thresholds. The persistent gap between the two curves suggests that the detector is highly effective at recognizing fall instances under the standard IoU threshold, while precise bounding-box localization under stricter overlap criteria remains more challenging. Overall, the convergence of both curves indicates stable training and strong detection performance of the YOLO model.

The YOLO classification results for the three fall directions were highly consistent under the adopted experimental setting, which supports the good separability of these classes in the analyzed infrared data.

### 4.4. Discussion

The comparative analysis of YOLO and GCN highlights distinct but complementary strengths. The GCN model achieves very strong class discrimination, as reflected by the high validation F1-score approaching 1.00 and by the normalized confusion matrix, where the diagonal values reach 0.97 for *Forwards*, 0.98 for *Side*, and 1.00 for *Other*. These results indicate that graph-based modeling is highly effective for separating fall-direction classes when reliable skeletal data are available. In contrast, YOLO offers direct detection and localization from infrared frames, which makes it attractive for real-time deployment scenarios. However, the validation curves show that recall rises earlier and remains higher than precision during a substantial part of training, suggesting that the detector is highly sensitive to fall events but may still generate false positives before full convergence.

This interpretation is further supported by the detector-specific metrics. In particular, mAP@0.5 increases rapidly and remains close to 1.0 during the later stage of training, whereas mAP@0.5:0.95 stabilizes at a lower level of approximately 0.75–0.79. This gap suggests that YOLO is highly effective at recognizing fall instances under the standard IoU criterion, while precise localization under stricter overlap thresholds remains more challenging. Therefore, the quantitative comparison shows a clear trade-off: YOLO provides fast end-to-end detection and strong recall, whereas GCN provides more stable class-level discrimination and stronger motion-pattern separability.

When interpreted against the currently available FIR-Human-based literature, our results should be viewed alongside the FIR fall detection pipeline of Gutiérrez et al., who reported 95.83% sensitivity, 96.92% specificity, and 96.63% accuracy for their best configuration. However, this comparison should be made with caution because the target formulation, model pipeline, and evaluation protocol differ from those used in the present work [39].

From a methodological perspective, our results contribute to the ongoing debate within the pattern recognition community regarding the relative merits of dense, pixel-level representations versus structured, graph-based motion models. While YOLO demonstrates strong performance in raw image domains, GCN provides a natural framework for encoding relational dependencies in skeletal data and is comparatively less dependent on raw appearance cues. Within the available synchronized multi-view infrared/skeleton setting, this makes the graph-based approach less sensitive to viewpoint-related appearance variation, although dedicated cross-view experiments would still be required to confirm full viewpoint robustness. This comparative analysis may inform the design of future models for action recognition, anomaly detection, or other sequence-based pattern recognition tasks beyond fall detection. This perspective is also consistent with recent work on gait and motion-pattern recognition [42], which underlines the importance of strong quantitative evidence in human movement analysis.

#### Limitations and Future Work

Despite the promising results, several limitations of this study should be acknowledged.

First, the experiments were conducted on a relatively small and demographically limited dataset collected in a controlled laboratory environment. Although the FIR-Human dataset includes five volunteers, only four contributed fall recordings used for fall-direction analysis, while one participant performed only non-fall activities. This limited subject diversity constrains the generalizability of the reported results.

A key limitation of the adopted GCN evaluation protocol is that the train/validation/test split was performed after converting recordings into frame-level graph samples. As a result, temporally adjacent or otherwise highly similar frames from the same recording may be distributed across different subsets, introducing a risk of information leakage and potentially leading to overly optimistic performance estimates. Therefore, the reported GCN results should be interpreted with caution as evidence of discriminative capability within the adopted frame-level protocol rather than as a definitive estimate of sequence-independent generalization. This issue is further compounded by the lack of subject-independent evaluation, as samples from the same individuals may appear across training, validation, and test sets, potentially overestimating generalization to unseen subjects.

Another limitation concerns the reliance on a single sensing modality, namely, infrared imaging with synchronized skeletal annotations. The study does not explore whether multimodal fusion could further improve robustness. In addition, the evaluation does not include realistic home environments, severe occlusions, cluttered scenes, or significant viewpoint variation, all of which may affect fall detection performance in practical deployments.

From a modeling perspective, the current GCN formulation employs a fully connected graph topology. While this design facilitates global message passing between all joints and simplifies the baseline architecture, it does not explicitly encode the anatomical structure of the human body. As a result, the model is less interpretable from a biomechanical perspective than approaches based on natural skeletal connections.

Finally, the study does not include a dedicated analysis of computational complexity, runtime efficiency under embedded constraints, or full edge-deployment feasibility. In addition, no repeated-run statistical analysis or confidence intervals were reported, which limits the ability to assess result stability and statistical significance.

Future work should address these limitations by introducing subject-independent and sequence-level evaluation protocols, exploring anatomically informed graph structures, and incorporating multimodal data fusion. Furthermore, larger and more diverse datasets, more realistic evaluation settings, and deployment-oriented benchmarking will be essential to validate real-world applicability.

## 5. Conclusions

This study presents a comparative analysis of graph convolutional networks (GCNs) and the YOLOv8 algorithm for human fall detection using infrared imaging. The experiments highlight that both approaches provide valuable but complementary strengths. GCN effectively models relational dependencies within skeletal data, which allowed it to achieve very high classification performance under the adopted evaluation protocol. Its graph-based representation makes it a promising tool for fine-grained human activity analysis, particularly when reliable skeletal input is available. However, its dependence on accurate skeletal data extraction and the computational cost of preprocessing represent notable limitations that may hinder deployment in less controlled environments.

YOLOv8, in contrast, proved effective in real-time detection directly from infrared video frames. Its speed and ability to localize subjects without the need for explicit skeletal reconstruction make it highly suitable for time-critical monitoring scenarios. Nonetheless, its reliance on bounding-box predictions makes it less sensitive to subtle postural changes, and its performance can degrade under occlusion or camera viewpoint shifts. These trade-offs suggest that neither approach alone fully addresses the challenges of robust, real-world fall detection.

For the research community, the presented comparative study provides a practical benchmark of two widely used artificial intelligence approaches applied to infrared-based fall detection. Researchers in healthcare monitoring, human–robot interaction, and intelligent surveillance can benefit from understanding the complementary capabilities of these models. In particular, our findings motivate future investigation of a hybrid decision-level framework that combines the fast localization strengths of YOLO with the motion-structure reasoning of GCN. In such a pipeline, YOLO could first localize the subject and identify candidate fall events directly from infrared frames, while GCN could analyze the corresponding skeletal motion patterns to refine fall-direction classification. Two example decision-level fusion strategies may be considered in future work. First, a confidence-weighted fusion scheme could combine the detector confidence from YOLO with the class confidence from GCN, for example, by assigning a higher weight to YOLO when localization is strong and a higher weight to GCN when the skeletal classification is temporally consistent. Second, a short-window temporal voting strategy could aggregate successive frame-level decisions over time so that isolated false alarms would be suppressed while persistent fall-related evidence would strengthen the final decision. Although this framework is not implemented in the present study, the comparative results reported here provide a practical rationale for its future investigation.

Future work should focus on several directions. First, larger and more diverse datasets are necessary to validate generalization across different populations, environments, and camera settings. Second, optimization of computational pipelines is needed to ensure scalability and real-time deployment in embedded or edge devices. Third, exploring multimodal data sources—such as integrating infrared imaging with depth sensors, WiFi-based signals, or wearable devices—may further reduce false alarms and improve robustness in cluttered or dynamic environments. Finally, ethical and practical considerations such as user privacy, system interpretability, and integration with assistive robotics should be addressed to enable real-world adoption.

In summary, while this study demonstrates the effectiveness of GCN and YOLO in infrared-based fall detection, its broader contribution lies in showing that the future of fall detection systems will likely depend on hybrid and multimodal solutions that balance accuracy, efficiency, and practicality. Importantly, the insights gained from this comparison are not limited to fall detection. The analysis of trade-offs between YOLO and GCN architectures may benefit researchers addressing broader challenges in pattern recognition, such as gesture recognition, human–robot interaction, or anomaly detection in video data.

## Figures and Tables

**Table 1:** List of main body joints.

Corresponding Number	Body Joint
1	Top of the head
2	Front of the head
3	Left arm
4	Left elbow
5	Left wrist
6	Right arm
7	Right elbow
8	Right wrist
9	Neck
10	Left hip
11	Right hip
12	Left knee
13	Left ankle
14	Left heel
15	Left toe
16	Right knee
17	Right ankle
18	Right heel
19	Right toe

**Table 2:** FIR-Human dataset summary (key properties used in this study).

Property	Value
Modality	Far-infrared video + synchronized 2D/3D joints
Resolution/FPS	480×640/23.98 FPS
Joints	19 main body joints (2D + 3D)
Subjects	5 (4 male, 1 female); BMI 16–24; ages 25–56
Environment	Laboratory; varying thermal conditions (16–31 °C)
Classes	27 total (26 daily activities + falls)
Views	Multi-view (four positions; frontal/rear/side perspectives)

**Table 3:** GCN architecture and training hyperparameters used in this study.

Setting	Value
Input graph	N=19 nodes (joints), fully connected; per-frame graphs
Node features	3D joint coordinates (x,y,z)
Classes	4 (*Forwards*, *Backwards*, *Side*, *Other*)
GCN backbone	4 × CNConv with residual (skip) connections
Channels	3→32→64→128→256
Activation/normalization	ReLU + BatchNorm after each GCN layer
Pooling	Global mean pooling
Classifier head	MLP: 256→128→64→4
Dropout	p=0.5 (before/within MLP head)
Loss	Weighted cross-entropy
Class weights	[1.0,2.0,3.0,1.0] (Fwd, Bwd, Side, Other)
Optimizer	Adam
Learning rate	$1\times 10^{-3}$
Weight decay	$1\times 10^{-4}$
Batch size	32 graphs
LR schedule	StepLR: step size 20 epochs, γ=0.5
Gradient clipping	Max norm 2.0
Training length	Up to 200 epochs; early stopping (patience = 10, val accuracy)
Augmentation	Random edge removal (edge perturbation) during training
Temporal handling	Frame-level GCN; sequence-level decision via aggregation
Inference aggregation	mean_softmax (default) or majority_vote over frames

**Table 4:** YOLOv8 training and inference settings used in this study (Ultralytics run configuration).

Setting	Value
Task/mode	Detection/training
Model variant	YOLOv8m (model = yolov8m.pt)
Pretrained initialization	pretrained = true
Epochs/batch size	150/16
Input image size	imgsz = 640
Seed/deterministic	seed = 0, deterministic = true
Mixed precision	amp = true
Optimizer	optimizer = auto
Base LR/final LR factor	lr0 = 0.01, lrf = 0.01
Momentum/weight decay	momentum = 0.937, weight_decay = 0.0005
warmup	warmup_epochs = 3.0, warmup_momentum = 0.8, warmup_bias_lr = 0.1
Augmentation (HSV)	hsv_h = 0.015, hsv_s = 0.7, hsv_v = 0.4
Augmentation (geom.)	translate = 0.1, scale = 0.5, degrees = 0.0, shear = 0.0, perspective = 0.0
Flips	fliplr = 0.5, flipud = 0.0
Mosaic/MixUp/Copy–paste	mosaic = 1.0 (disabled last 10 epochs: close_mosaic = 10); mixup = 0.0; copy_paste = 0.0
NMS IoU/confidence	iou = 0.7; conf = null (Ultralytics default)

## Data Availability

We used the dataset available on the IEEE Dataset portal [5].
